# The first report of adolescent TAFRO syndrome, a unique clinicopathologic variant of multicentric Castleman’s disease

**DOI:** 10.1186/1471-2431-14-139

**Published:** 2014-06-02

**Authors:** Ikuko Kubokawa, Akihiro Yachie, Akira Hayakawa, Satoshi Hirase, Nobuyuki Yamamoto, Takeshi Mori, Tomoko Yanai, Yasuhiro Takeshima, Eiryu Kyo, Goichi Kageyama, Hiroshi Nagai, Keiichiro Uehara, Masaru Kojima, Kazumoto Iijima

**Affiliations:** 1 Department of Pediatrics, Kobe University Graduate School of Medicine, 7-5-2 Kusunoki-Cho, Chuo-ku, Kobe 650-0017, Japan; 2 Department of Pediatrics, School of Medicine, Institute of Medical, Pharmaceutical and Health Sciences, Kanazawa University, 13-1 Takaramachi, Kanazawa 920-8641, Japan; 3 Department of Pediatrics, Nishiwaki Municipal Hospital, 652-1 Shimo-toda, Nishiwaki 677-0043, Japan; 4 Department of Rheumatology, Kobe University Graduate School of Medicine, 7-5-2 Kusunoki-Cho, Chuo-ku, Kobe 650-0017, Japan; 5 Department of Dermatology, Kobe University Graduate School of Medicine, 7-5-2 Kusunoki-Cho, Chuo-ku, Kobe 650-0017, Japan; 6 Department of Diagnostic Pathology, Kobe University Hospital, 7-5-2 Kusunoki-Cho, Chuo-ku, Kobe 650-0017, Japan; 7 Department of Diagnostic and Anatomic Pathology, Dokkyo Medical University School of Medicine, 880 Kitakobayashi, Mibu-machi, Shimotsuga-gun, Tochigi 321-0293, Japan

**Keywords:** Thrombocytopenia, Anasarca, reticulin Fibrosis of the bone marrow, Renal dysfunction, Organomegaly, Tocilizumab, IL-6, VEGF, Neopterin, Soluble TNF-receptors

## Abstract

**Background:**

TAFRO syndrome is a unique clinicopathologic variant of multicentric Castleman’s disease that has recently been identified in Japan. It is characterized by a constellation of symptoms: Thrombocytopenia, Anasarca, reticulin Fibrosis of the bone marrow, Renal dysfunction and Organomegaly (TAFRO). Previous reports have shown that affected patients usually respond to immunosuppressive therapy, but the disease sometimes has a fatal course. TAFRO syndrome occurs in the middle-aged and elderly and there are no prior reports of the disease in adolescents. Here we report the first adolescent case, successfully treated with anti-IL-6 receptor antibody (tocilizumab, TCZ) and monitored with serial cytokine profiles.

**Case presentation:**

A 15-year-old Japanese boy was referred to us with fever of unknown origin. Whole body computed tomography demonstrated systemic lymphadenopathy, organomegaly and anasarca. Laboratory tests showed elevated C-reactive protein and hypoproteinemia. Bone marrow biopsy revealed a hyperplastic marrow with megakaryocytic hyperplasia and mild reticulin fibrosis. Despite methylprednisolone pulse therapy, the disease progressed markedly to respiratory distress, acute renal failure, anemia and thrombocytopenia. Serum and plasma levels of cytokines, including IL-6, vascular endothelial growth factor, neopterin and soluble tumor necrosis factor receptors I and II, were markedly elevated. Repeated weekly TCZ administration dramatically improved the patient’s symptoms and laboratory tests showed decreasing cytokine levels.

**Conclusion:**

To our knowledge, this is the first report of TAFRO syndrome in a young patient, suggesting that this disease can occur even in adolescence. The patient was successfully treated with TCZ. During our patient’s clinical course, monitoring cytokine profiles was useful to assess the disease activity of TAFRO syndrome.

## Background

TAFRO syndrome is a unique clinicopathologic variant of multicentric Castleman’s disease that has recently been identified in Japan [1]. The syndrome is characterized by a constellation of symptoms: Thrombocytopenia, Anasarca, reticulin Fibrosis of the bone marrow, Renal dysfunction and Organomegaly (TAFRO). Although elevated levels of interleukin-6 (IL-6) and vascular endothelial cell growth factor (VEGF) are seen in the serum and effusions of patients with TAFRO syndrome, the pathogenesis of the disease remains obscure [1]. Previous reports [2-6] have shown that patients respond to immunosuppressive therapy, but the disease has resulted in a fatal outcome in some patients [5,6]. This disease occurs in the middle-aged and elderly [1]; no case of TAFRO syndrome in adolescence has been reported to date.

Here we report the case of a 15-year-old Japanese boy with TAFRO syndrome successfully treated with anti-IL-6 receptor antibody (tocilizumab, TCZ) and monitored with serial precise cytokine profiles. This is the first report of this disease in an adolescent.

## Case presentation

### Clinical course

**Figure 1:**
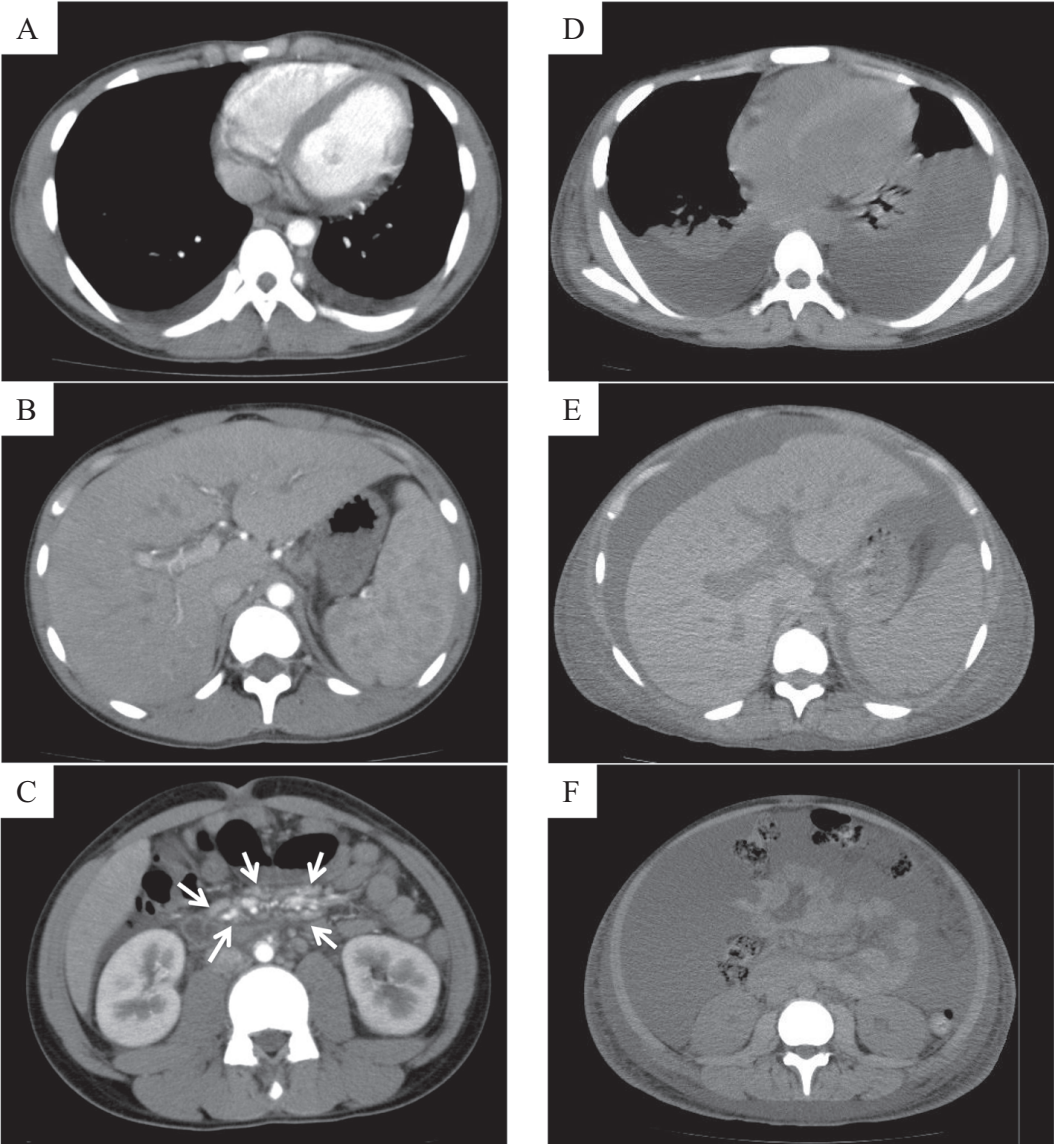
**Imaging study.** The patient had pleural effusion **(A)** and severe hepatosplenomegaly **(B)**. Multiple lymph node enlargements were observed in the mesentery and in the paraaortic lymph nodes at admission (arrows in white) **(C)**. After two weekly TCZ infusions, systemic lymphadenopathy, hepatosplenomegaly and renal enlargement improved. However, pleural fluid, ascites and subcutaneous edema worsened **(D–F)**.

**Figure 2:**
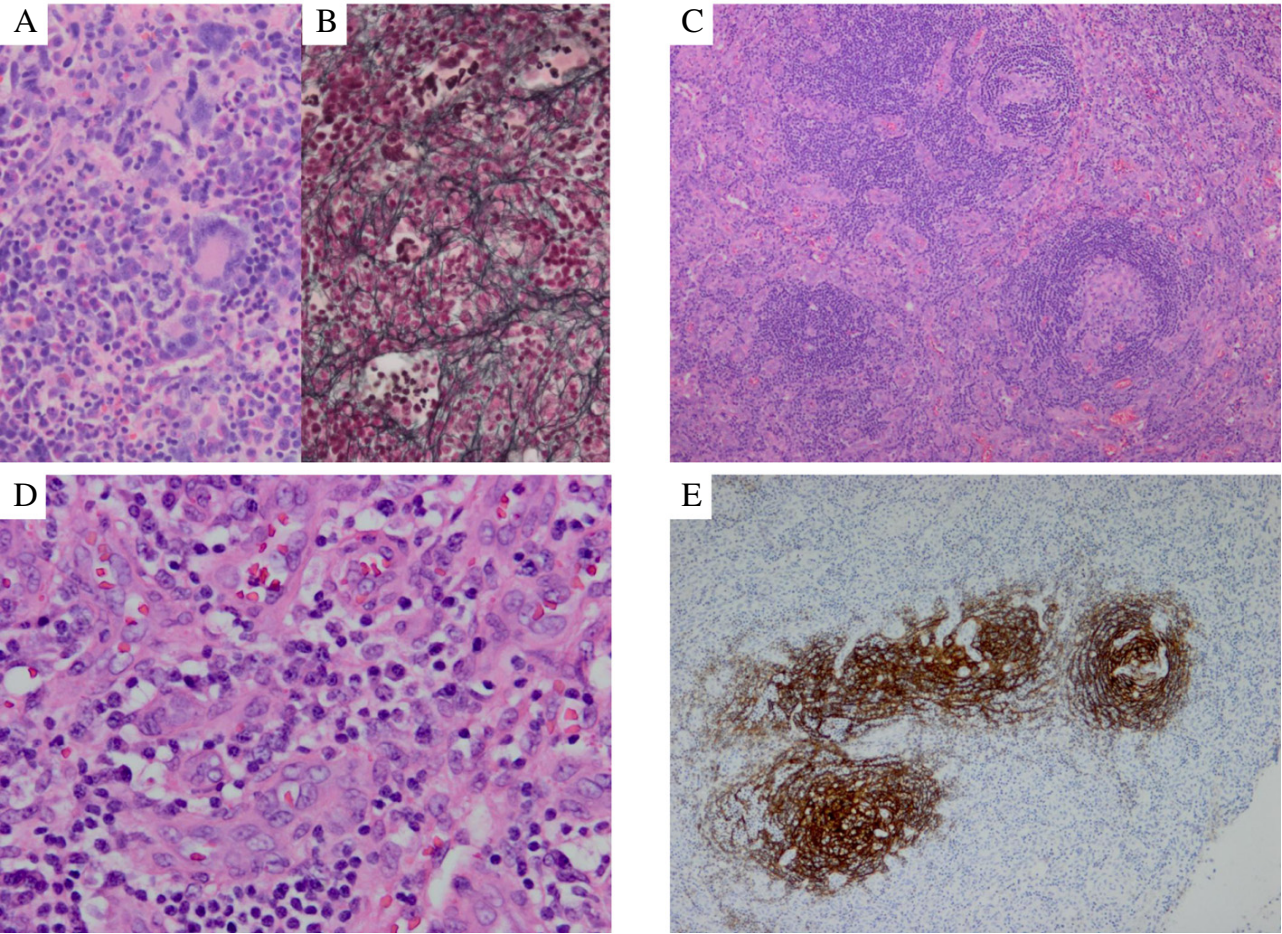
**Histopathological findings of the bone marrow (A, B) and lymph nodes (C–E). (A)** Hematoxylin and Eosin stain × 200. Bone marrow biopsy showed hypercellular marrow with increased numbers of megakaryocytes, including micro- and multi-separated nuclear megakaryocytes and megaloblastic change. **(B)** Silver stain × 200. Silver stain showed mild reticulin fibrosis. **(C)** Hematoxylin and Eosin stain × 200. A high-power field in the lymph node showed scattered lymphoid follicles with atrophic germinal centers, enlarged follicular dendritic cells, surrounding concentric rings of small lymphocytes, and penetrating vessels. **(D)** Hematoxylin and Eosin stain × 200. The interfollicular area was characterized by the proliferation of highly dense endothelial vessels and moderate numbers of mature plasma cells. **(E)** CD21 immunostain × 200. Immunostaining for CD21 showed tight/concentric and expanded/disrupted pattern of follicular dendritic cells. These findings were compatible with mixed-type Castleman’s disease.

A 15-year-old Japanese boy was referred to us with fever of unknown origin of 2 weeks’ duration. He had a systolic murmur and hepatosplenomegaly. The patient’s superficial lymph nodes were swollen, with a maximum diameter of 3.0 cm. Laboratory tests showed elevations in C-reactive protein (CRP; 17.8 mg/dL), soluble IL-2 receptor (2,467 IU/mL), lactate dehydrogenase (511 IU/L) and D-D dimer (5.6 μg/mL). The patient had decreased total protein (5.1 g/dL), albumin (1.8 g/dL), immunoglobulin G (729 mg/dL) and cholinesterase (48 IU/L). Complete blood cell count and serum levels of liver enzymes, blood urea nitrogen and creatinine (Cr) were within normal range. Urinalysis showed mild proteinuria of 0.4 mg/mg ∙ Cr without hematuria. Enhanced whole body computed tomography demonstrated systemic lymphadenopathy, hepatosplenomegaly, renal enlargement and anasarca (Figure 1A–C).Bone marrow biopsy revealed a hyperplastic marrow with megakaryocytic hyperplasia (Figure 2A) and mild reticulin fibrosis (Figure 2B). An 18-fluoro-deoxyglucose (FDG) positron emission tomography scan showed weak FDG uptake by the bilateral cervical and inguinal lymph nodes and spleen. Biopsies of the lymph nodes showed scattered lymphoid follicles with atrophic germinal centers and enlarged follicular dendritic cells, with surrounding concentric rings of small lymphocytes and penetrating vessels (lollipop-like appearance; Figure 2C). The interfollicular area was characterized by prominent vascularity and moderate numbers of mature plasma cells (Figure 2D). Immunological studies showed decreased numbers of B-cells and CD57+ T-cells in the germinal centers. Immunostaining for CD21 demonstrated tight/concentric and expanded/disrupted patterns of follicular dendritic cells (Figure 2E). These findings were compatible with the mixed type of Castleman’s disease.

Autoantibodies, serum M-protein and urine Bence-Jones protein were not detected in this patient. Blood culture and quantitative PCR examinations for cytomegalovirus, Epstein-Barr virus, human herpes virus type 8 (HHV-8) and human immunodeficiency virus (HIV) were all negative. There were no significant pathological findings of malignancy in lymph node or liver biopsies.Despite treatment with antibiotics and albumin, the disease progressed markedly to respiratory distress, oliguric renal failure, anemia and thrombocytopenia. Methylprednisolone pulse therapy at a dose of 1000 mg/day for 3 consecutive days was initiated on day 10 after admission, but the patient’s fever persisted and his CRP remained elevated. The patient was diagnosed with multicentric Castleman’s disease and treatment was initiated with weekly TCZ at a dose of 8 mg/kg, high dose intravenous immunoglobulin and 80 mg of prednisolone (PSL) daily. Weekly TCZ dramatically improved the patient’s symptoms and laboratory findings. However, anasarca persisted (Figure 1D–F). With removal of ascites, anasarca gradually disappeared.

**Figure 3:**
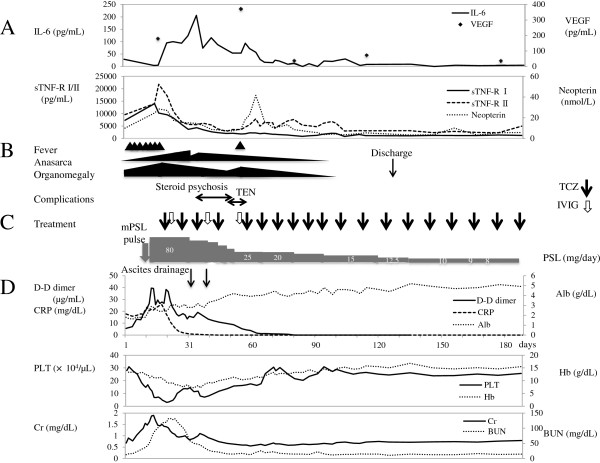
**Patient’s clinical course. A**: Cytokine profiles; **B**: Clinical symptoms and complications; **C**: Treatment; **D**: Laboratory data. IL-6: interleukin-6; IVIG: intravenous injection of immunoglobulin (⇩); mPSL: methylprednisolone; PSL: prednisolone; sTNF-R: soluble tumor necrosis factor receptor; TCZ: tocilizumab (↓); TEN: toxic epidermal necrolysis; VEGF: vascular endothelial cell growth factor.

One month after the initiation of TCZ therapy, the patient was forced to decrease his PSL dose because of steroid psychosis. In addition, he developed blisters over his entire body and was forced to discontinue treatment with TCZ because drug reaction or viral infection was suspected. Paraneoplastic pemphigus and pemphigoid, which are reported complications of Castleman’s disease [7-9], were ruled out by negative antibody and immunofluorescence testing. We diagnosed the patient’s skin lesions as toxic epidermal necrolysis by pathology, but were unable to determine the cause.After treatment for multicentric Castleman’s disease was discontinued, the patient’s clinical symptoms reappeared with elevated cytokine levels. Restarting weekly TCZ resulted in improvement in these findings (Figure 3). During this treatment, the patient’s skin lesions also resolved. After cytokine levels normalized 4 months after admission, the patient was discharged. One year after disease onset, the patient continued treatment with 5 mg PSL daily and TCZ every 3 weeks without any signs of recurrence. His clinical symptoms completely matched those of TAFRO syndrome.

### Cytokine profile of serum, plasma and ascites

In the acute phase of the disease, the patient’s serum cytokine levels of IL-6, IL-7, IL-10, IL-12p70, IL-15, IL-16, soluble tumor necrosis factor receptors I and II (sTNF-R I/II), VEGF, neopterin, interferon gamma-induced protein 10 (IP-10), macrophage inflammatory protein 1β (MIP-1β), eotaxin-3 and monocyte chemoattractant protein 1 (MCP-1) were elevated (Table 1). After initiation of TCZ therapy, serum IL-6 levels increased because of IL-6 receptor blocking by TCZ (Figure 3). After repeated TCZ infusions, most of the serum and plasma cytokine/chemokine levels decreased, including IL-6. When TCZ therapy was discontinued because of steroid psychosis and toxic epidermal necrolysis, cytokine levels transiently increased. After restarting TCZ therapy, cytokine levels decreased once more (Figure 3).

**Table 1:** Cytokine profiles of serum, plasma and ascites

		**Serum**			**Ascites**		**Reference range**
		**On admission**	**Day 17 (before weekly TCZ)**	**Day 321**	**Day 29**	**Day 36**	**Serum**
IL-1α	pg/mL	<1.37	<1.37	<1.37	<1.37	<1.37	0.29–62.1
IL-1β	pg/mL	<0.640	<0.640	<0.640	1.620	0.826	0.11–24.3
IL-2	pg/mL	<1.78	<1.78	<1.78	6.84	2.00	0.22–2.68
IL-4	pg/mL	0.542	0.376	0.642	2.90	3.84	N.A.
IL-5	pg/mL	<3.2	<3.2	<3.2	6.70	5.72	0.11–0.62
IL-6	pg/mL	29	5.0	5.0	4400	4700	<3
IL-7	pg/mL	146	54.6	89.0	17.6	20.8	0.37–2.78
IL-8	pg/mL	230	348	34.8	>798	522	1.48–1720
IL-10	pg/mL	3.28	23.2	1.36	8.00	10.4	0.06–3.08
IL-12p70	pg/mL	3.40	3.16	3.20	5.30	31.0	0.26–0.38
IL-12/IL-23p40	pg/mL	488	49.2	330	45.2	93.6	13.1–159
IL-13	pg/mL	6.98	8.28	8.90	17.0	12.0	0.60–2.78
IL-15	pg/mL	11.6	36.8	4.74	34.8	43.6	0.56–3.01
IL-16	pg/mL	1054	1266	628	366	610	24–137
IL-17A	pg/mL	<17.7	<17.7	<17.7	<17.7	<17.7	0.28–4.87
IL-18	pg/mL	360	230	182	75.0	80.0	<500
IFN-γ	pg/mL	14.8	<5.12	22.2	19.3	10.7	0.64–14.4
TNF-α	pg/mL	<5	<5	N.D.	<5	<5	<5
sTNF-RI	pg/mL	7300	10200	1230	8000	4800	484–1407
sTNF-RII	pg/mL	9600	21800	3450	6000	4200	829–2262
VEGF	pg/mL	N.D.	178*	16.4*	1860	1210	<38.3*
Neopterin	nmol/L	10.0	28.5	4.00	16.7	15.5	<5
GM-CSF	pg/mL	<3.8	<3.8	<3.8	<3.8	<3.8	0.18–0.18
IP-10	pg/mL	>2000	>2000	928	1200	1440	28.5–237
MIP-1α	pg/mL	87.6	56.0	<55.2	71.6	77.2	12–202
MIP-1β	pg/mL	628	480	452	166	188	7.29–95.2
Eotaxin	pg/mL	76.8	<48.8	228	85.6	162	19.0–145
Eotaxin-3	pg/mL	61.2	68.0	76.0	404	624	7.63–8.73
TARC	pg/mL	132	58.8	860	30.2	33.2	5.39–70.0
MCP-1	pg/mL	>1508	528	632	>1508	>1508	75.7–205
MCP-4	pg/mL	180	47.2	209	36.2	39.2	11.2–117
MDC	pg/mL	1960	330	2740	<320	374	606–3249

IL: interleukin; IFN: interferon; TNF: tumor necrosis factor; sTNF-RI: soluble tumor necrosis factor receptor type I; sTNF-RII: soluble tumor necrosis factor receptor type II; VEGF: vascular endothelial cell growth factor; GM-CSF: granulocyte macrophage colony-stimulating factor; IP-10: interferon gamma-induced protein 10; MIP: macrophage inflammatory protein; TARC: thymus and activation regulated chemokine; MCP: monocyte chemoattractant protein; MDC: macrophage-derived chemokine; N.A: not available; N.D: not determined: *: values derived from plasma.

Aspiration of ascites was performed twice with a total volume of 11.2 L removed. IL-6 and VEGF levels in the ascitic fluid were extremely high (Table 1).

### Cytokine and chemokine determination

Serum and plasma concentrations of IL-6, IL-18, tumor necrosis factor α (TNF-α), sTNF-R I/II, VEGF and neopterin were determined by using the following enzyme-linked immunosorbent assay (ELISA) kits: neopterin (IBL, Hamburg, Germany); IL-6, TNF-α, sTNF-R I/II and VEGF (R&D Systems Inc., Minneapolis, MN, USA); and IL-18 (MBL, Nagoya, Japan). Other cytokines/chemokines were determined by electrochemiluminescence immunoassay (MSD, Rockville, MD, USA).

## Discussion

Multicentric Castleman’s disease is thought to comprise several disease entities, including idiopathic and secondary multicentric Castleman’s disease in conditions such as POEMS syndrome, autoimmune disease-associated lymphadenopathy and malignant lymphoma [10,11]. In contrast to its prevalence in Western countries [11,12], multicentric Castleman’s disease associated with HIV and/or HHV-8 is uncommon in Japan, where the disease usually demonstrates a relatively chronic course. Kojima et al. classified Japanese multicentric Castleman’s disease into two subtypes on the basis of clinicopathological findings: (1) idiopathic plasmacytic lymphadenopathy with polyclonal hyperimmunoglobulinemia (IPL type) and (2) non-IPL type, which is atypical multicentric Castleman’s disease characterized by mixed-type or hyaline vascular-type histology and a high incidence of massive effusion and autoimmune disease [13]. IPL is considered a homogeneous disease entity, whereas non-IPL type is a heterogeneous cluster of disease entities [13,14].

Recently Takai et al. reported three cases that shared a constellation of clinical symptoms: thrombocytopenia, anasarca, fever, reticulin fibrosis of the bone marrow and organomegaly. These symptoms were tentatively given the clinical name “TAFRO syndrome” to describe the new disease concept [6]. In 2013, Japanese national meetings were held to define TAFRO syndrome more clearly as a systemic inflammatory disease characterized by a constellation of symptoms: thrombocytopenia, anasarca, reticulin fibrosis of the bone marrow, renal dysfunction and organomegaly [1]. Other clinical findings include anemia, immunologic disorder and rarely polyclonal hyper-γ-globulinemia [1]. As a subtype of non-IPL, TAFRO syndrome is characterized histologically as mostly a mixed type of Castleman’s disease, with an abnormal follicular dendritic cell network [1]. TAFRO syndrome is considered a novel clinical entity belonging to systemic inflammatory disorders and featuring immunological abnormality beyond the ordinary spectrum of multicentric Castleman’s disease [1].

It is thought that the pathogenesis of TAFRO syndrome might be associated with a strong hypercytokine storm, including IL-6 and VEGF [1,5]. In our patient, serum and plasma levels of not only IL-6 and VEGF but also of other cytokines/chemokines were markedly elevated (Table 1). It is not clear how these cytokines/chemokines are associated with this disease. Further studies are needed to clarify the roles of these cytokines/chemokines. It is interesting that the levels of IL-6 and VEGF in the ascitic fluid of our patient were markedly higher than the levels in his serum and plasma. One previous case has been reported of a patient with severe anasarca and markedly elevated IL-6, suggesting systemic inflammation of the serosa [3].

It is thought that the thrombocytopenia seen in TAFRO syndrome might be caused by an immune-mediated mechanism and can be overcome by anti-inflammatory therapy [2-4]. The mechanism of renal failure in patients with TAFRO syndrome is not clear because histological examinations of kidneys have not been reported in this disease. Previous studies have reported that patients with Castleman’s disease manifest renal symptoms such as nephrotic syndrome and acute renal failure and that histologic findings were heterogeneous, including various glomerular lesions, thrombotic microangiopathy-like lesions, interstitial nephritis and amyloidosis [15,16]. In our case, urinalysis showed mild proteinuria and mildly elevated urinary N-acetyl-beta-D-glucosaminidase and β2-microglobulin without fractural red blood cells in peripheral blood before the onset of renal failure. These findings suggest that the kidney pathophysiology in this case was interstitial nephritis rather than glomerular nephritis, thrombotic microangiopathy or amyloidosis.

Eight cases of TAFRO syndrome have previously been reported [2-6]. All cases including ours are summarized in Table 2. This disease generally occurs in the middle-aged and elderly [1-6]. There are no adolescent cases reported to date, and this is the first case of a young patient with an apparent diagnosis of TAFRO syndrome. All patients were treated with steroids and some improved with the addition of cyclosporine A [2,6] or TCZ [3,4] with rituximab therapy [3]. Unfortunately, three of the eight patients died [5,6], indicating that this syndrome sometimes results in a fatal outcome in spite of treatment.

**Table 2:** Clinical features of TAFRO syndrome in eight previously reported cases and in our case

**Case no.**	**Age/sex**	**Thrombocytopenia (PLT ×10**^**4**^**/μl)**	**Anasarca**	**Reticulin fibrosis of the bone marrow**	**Renal dysfunction**	**Organomegaly**	**Hb (g/dl)**	**IgG (mg/dl)**	**Serum IL-6 (pg/ml) (normal range)**	**Plasma VEGF (pg/ml) (normal range)**	**Lymph node biopsy**	**Treatment**	**Outcome**	**Ref**
1	47/F	1.5	+	+	No data	+	10.9	1046	No data	285 (<115)	No data	CHOP, PSL	Survival	[6]
2	56/M	1.9	+	+	+	+	10.7	863	7.2 (<4)	31 (<115)	No data	PSL, IVIG, CyA	Survival	
3	49/M	1.0	+	+	No data	+	11.7	1057	64.9 (<4)	104 (<115)	HV-type of CD	PSL, IVIG	Death (MOF)	
4	49/F	1.7	+	+	+	+	6.2	2611	11 (No data)	330 (No data)	unclear	DEX, PSL, CyA	Survival	[2]
5	43/F	<3.0	+	No data	+	+	12.6	965	45.6 (<4)	665 (<115)	HV-type of CD	mPSL, PSL, RTX, TCZ	Survival	[3]
6	47/F	3.9	+	+	+	+	9.1	1426	21.9 (No data)	No data	PC-type of CD	PSL, mPSL, TCZ	Survival	[4]
7	57/F	1.3	+	-	+	+	7.1	1860	65.5 (No data)	91 (No data)	unclear	mPSL, PSL, CHOEP	Death (Sepsis)	[5]
8	73/M	2.4	+	+	+	+	7.6	7080	49 (No data)	25 (No data)	Mixed type of CD	mPSL, PSL	Death (MOF)	
Current case	15/M	3.0	+	+	+	+	7.2	729	29 (<3)	178 (<38.3)	Mixed type of CD	mPSL, PSL, TCZ, IVIG	Survival	

CD: Castleman’s disease; CHOEP: cyclophosphamide, doxorubicin, vincristine, etoposide and prednisolone; CHOP: cyclophosphamide, doxorubicin, vincristine and prednisolone; CyA: cyclosporine A; Hb: hemoglobin; HV: hyaline vascular type; IgG: immunoglobulin G; IL-6: interleukin-6; IVIG: intravenous immunoglobulin; MOF: multiple organ failure; mPSL: methylprednisolone; PC: plasma cell type; PLT: platelet; PSL: prednisolone; RTX: rituximab; TCZ: tocilizumab; VEGF: vascular endothelial cell growth factor; Ref: reference number.

There are no reports of TAFRO syndrome outside of Japan. However, we found one report of multicentric Castleman’s disease in a 4-year-old Hispanic girl with thrombocytopenia, anasarca and renal failure [17]. Although the authors diagnosed the patient with multicentric Castleman’s disease and did not comment on reticulin fibrosis of the bone marrow, her clinical characteristics were quite similar to those of our patient and to other cases of TAFRO syndrome. The question remains whether TAFRO syndrome should be classified as a distinct disease entity rather than as an atypical subtype of multicentric Castleman’s disease. All of the features of TAFRO syndrome can be seen in severe flares of multicentric Castleman’s disease. For example, anasarca and organomegaly are common [11], and thrombocytopenia and renal dysfunction are frequently reported in Castleman’s disease [10,14-17]. In contrast, reticulin fibrosis of the bone marrow occurs less frequently, but it is not specific for TAFRO syndrome or for multicentric Castleman’s disease. Establishing TAFRO syndrome as an independent disease entity remains controversial. Further investigation with clinical and pathological studies is necessary to establish TAFRO syndrome as a new syndromic disease.

## Conclusions

It is important to note that TAFRO syndrome occurs not only in adults, but also in adolescents. In the acute phase, TAFRO syndrome should be treated with rapid and strong immunosuppressive therapy including TCZ to prevent a fatal outcome. We believe that cytokine profiling is useful not only to assess the pathogenesis but also to monitor disease activity of this rare disease.

## Consent

Written informed consent was obtained from the patient’s parents for publication of this case report and any accompanying images. A copy of written consent is available for review by the Editor-in-Chief of this journal.

## Abbreviations

Cr: Creatinine; CRP: C-reactive protein; HHV-8: Human herpes virus type 8; HIV: Human immunodeficiency virus; IL-6: Interleukin-6; PSL: Prednisolone; ELISA: Enzyme-linked immunosorbent assays; sTNF-R: Soluble tumor necrosis factor receptor; IP-10: Interferon gamma-induced protein 10; MIP-1β: Macrophage inflammatory protein 1β; MCP-1: Monocyte chemoattractant protein 1; TAFRO: Thrombocytopenia, Anasarca, reticulin Fibrosis of the bone marrow, Renal dysfunction and Organomegaly; TCZ: Tocilizumab; VEGF: Vascular endothelial cell growth factor.

## Competing interests

KI received research grants from Chugai Pharma. The other authors declare no competing interests.

## Authors’ contributions

IK treated and managed this patient, drafted the initial manuscript and approved the final manuscript as submitted. AY analyzed cytokine levels of IL-6 IL-18, TNF-α, sTNF-R I/II and neopterin, interpreted cytokine profiling and coordinated and supervised the management of the patient. AH, SH, NY, TM, TY, YT and GK supervised the management of the patient. EK initially treated this patient and referred him to us. HN performed pathological evaluation for the toxic epidermal necrolysis and supervised the management of the patient. KU and MK performed pathological evaluation for multicentric Castleman’s disease and TAFRO syndrome and supervised the management of the patient. KI supervised the management of the patient and critically reviewed the manuscript. All authors read and approved the final manuscript as submitted.

## Pre-publication history

The pre-publication history for this paper can be accessed here:

http://www.biomedcentral.com/1471-2431/14/139/prepub
